# Ambivalence about supervised injection facilities among community stakeholders

**DOI:** 10.1186/s12954-015-0060-3

**Published:** 2015-08-21

**Authors:** Carol Strike, Tara Marie Watson, Gillian Kolla, Rebecca Penn, Ahmed M. Bayoumi

**Affiliations:** Dalla Lana School of Public Health, University of Toronto, 155 College Street, Toronto, ON M5T 3M7 Canada; Social and Epidemiological Research, Centre for Addiction and Mental Health, 33 Russell Street, Toronto, ON M5S 2S1 Canada; Centre for Research on Inner City Health, Li Ka Shing Knowledge Institute, St. Michael’s Hospital, 30 Bond Street, Toronto, ON M5B 1W8 Canada; Department of Medicine, University of Toronto, Medical Sciences Building, 1 King’s College Circle, Toronto, ON M5S 1A8 Canada; Institute of Health Policy, Management, and Evaluation, University of Toronto, Health Sciences Building, 155 College Street, Suite 425, Toronto, ON M5T 3M6 Canada; Division of General Internal Medicine, St. Michael’s Hospital, 30 Bond Street, Toronto, ON M5B 1W8 Canada

**Keywords:** Supervised injection facilities, Public opinion, Telephone survey, Canada

## Abstract

**Background:**

Community stakeholders express a range of opinions about supervised injection facilities (SIFs). We sought to identify reasons for ambivalence about SIFs amongst community stakeholders in two Canadian cities.

**Findings:**

We used purposive sampling methods to recruit various stakeholder representatives (*n* = 141) for key informant interviews or focus group discussions. Data were analyzed using a thematic process. We identified seven reasons for ambivalence about SIFs: lack of personal knowledge of evidence about SIFs; concern that SIF goals are too narrow and the need for a comprehensive response to drug use; uncertainty that the community drug problem is large enough to warrant a SIF(s); the need to know more about the “right” places to locate a SIF(s) to avoid damaging communities or businesses; worry that a SIF(s) will renew problems that existed prior to gentrification; concern that resources for drug use prevention and treatment efforts will be diverted to pay for a SIF(s); and concern that SIF implementation must include evaluation, community consultation, and an explicit commitment to discontinue a SIF(s) in the event of adverse outcomes.

**Conclusions:**

Stakeholders desire evidence about potential SIF impacts relevant to local contexts and that addresses perceived potential harms. Stakeholders would also like to see SIFs situated within a comprehensive response to drug use. Future research should determine the relative importance of these concerns and optimal approaches to address them to help guide decision-making about SIFs.

## Findings

### Introduction

Public opinion is an important factor to consider in policy making [1], including drug policy [2, 3]. However, public opinion sometimes runs counter to scientific evidence [4]. This situation is reflected in studies showing that supervised injection facilities (SIFs), designed to reduce injection drug use-related health problems, remain controversial among some stakeholders (e.g., residents, business owners, politicians, and police). Despite evidence to the contrary, some stakeholders believe that SIFs promote initiation of injection drug use, endorse continued drug use at the expense of encouraging entry into drug treatment, and/or promote congregation of people who use and sell drugs which may lead to increased crime in the surrounding area [5, 6]. We previously reported findings showing that public support for SIFs in Ontario, Canada increased between 2003 and 2009 [7]. However, we also reported that the majority of the public continued to hold ambivalent opinions about SIF implementation [7].

In cities where some stakeholders are currently advocating for SIFs (e.g., New York, San Francisco, Toronto, Ottawa, and Montreal) [8–10], addressing public ambivalence may be important because decision makers are more likely to act when public opinion is supportive of policies [3]. In this brief report, we build upon and supplement previous analyses of ambivalent attitudes within the general public [7] with qualitative data analyses to identify potential underlying reasons for SIF ambivalence among community stakeholders.

### Methods, ethics and consent

In Toronto and Ottawa, we used purposive methods to recruit stakeholders (*n* = 141; 61 in Toronto and 80 in Ottawa) from varied sectors and groups: health care, addiction services, public health, law enforcement, fire and ambulance services, and housing and social services, as well as local residents and business representatives. To align with our analyses of public opinion data and in the interests of brevity, this sub-analysis of data focuses on these stakeholders. We also recruited people who use drugs (*n* = 95, including people who inject drugs, people who smoke drugs like crack cocaine, and those who use drugs in both ways) as part of the larger mixed-methods study. Their opinions about supervised consumption design preferences and SIF policy are reported elsewhere [11, 12]. In the study design, we proposed to recruit equal numbers from each city; however, during recruitment a large, multi-stakeholder, community group interested in public safety issues asked the investigators to conduct focus groups with them. Given that this group represented a similar range of stakeholders of interest for our study, we granted their request. This study was approved by the research ethics boards at St. Michael’s Hospital and the Centre for Addiction and Mental Health. We have previously provided more details about participant recruitment methods [12].

We used an iterative approach to data collection and thematic analyses informed by a grounded theory approach [13]. Members of the research team designed the interview and focus group guides which were pilot tested during initial consultations with participants. Refinements to question wording and new questions were added to the guides based on participant responses during these early interviews and focus groups. We conducted one-on-one key informant interviews (*n* = 26) and focus group discussions (*n* = 115 people) between December 2008 and January 2010. All participants provided informed consent, including permission to use anonymized quotes from their data, and were offered a $25 CAD honorarium. All interviews and focus groups were audio-recorded. During all interviews and focus groups, participants were asked questions about the following topics: perceived drug use in their communities; the benefits and drawbacks of SIFs; potential SIF locations and policies; and other approaches to address drug use.

Transcripts were stored and coded using NVivo 8 software. An analytic sub-team developed a codebook that contained key themes and subthemes of interest. Coding meetings were held with sub-team members where any coding discrepancies or suggestions for new codebook themes were discussed and resolved by group consensus.

### Results

In this analysis, we focus on seven reasons for ambivalence about SIFs. Sending the “wrong message” and risk of increased drug use and/or injecting were concerns expressed by some participants, but were typically done so in relation to outright dismissal or disagreement with SIFs, rather than ambivalence. Please see Table 1 for a summary of the reasons for ambivalence and supporting excerpts from the data. With the exception of the police in this study, who were uniformly opposed to SIFs [14], most participants voiced ambivalent opinions about SIF implementation: e.g., “I sit on the fence”, “I’m torn”, and “I’m up in the air with it”. Positive and negative SIF outcomes (e.g., reductions in HIV prevalence in the community; increases in drug-related crime around the facility) were positioned as crucial to the acceptance or rejection of SIFs, and stakeholder ambivalence was partially linked to a lack of knowledge about such outcomes and evidence. Citing lack of sufficient personal knowledge, some stakeholders refused to offer any opinions about SIFs, including a public official in Toronto who said, *“*I don’t feel like I have all of the information that I would need to make a sound recommendation.”

**Table 1:** Reasons for ambivalence about SIFS

Reason	Illustrative excerpts
1. Lack of personal knowledge of evidence about SIFs	*It’s easy just to say… well I wouldn’t want one because I’m going to have all these people wandering around downtown as high as kites. But then once you find out, well actually no, because this is set in place so that doesn’t happen… it could change people’s opinions, and they might be like, “Okay, well I can see the benefit of that then.” Knowledge can change everything.* (Toronto business owner)
2. Concern that SIF goals are too narrow and need to be located within a comprehensive response to drug use (“Comprehensive” defined with a range of suggestions)	*So there’s got to be more of a point to this, I think, than just a clean place for these drugs…The thing for us is that we might be able to convince half of these people to stop.* (Ottawa ambulance personnel)
	*I think we’re not serving people well if we just focus on their addiction, if we don’t also provide mental health services. … I think the community would be more receptive, from our drug strategy work, as long as harm reduction was connected to treatment options and housing and all of those other things… than if it’s just kind of seen as giving up on people, and just warehousing them in this little place downtown, not that that’s maybe the perception often I think.* (Ottawa community/service provider)
3. Uncertainty that the community drug problem is large enough to warrant SIF implementation	*We haven’t hit that crisis point, as Vancouver has hit… where they were doing it [injecting] in the daytime, around strollers and stuff. Get them off the street. We haven’t hit that point yet, so why would we even put all our money into that when we haven’t gotten there yet.* (Toronto ambulance personnel)
4. Need to know more about the so-called “right” places to locate SIFs to avoid damaging communities or businesses	*I struggle because [I] can see the potential benefits but an SIF would have a negative impact. The local [methadone maintenance treatment] clinic has created problems with thefts etc. [SIFs] would damage business.* (Toronto resident)
	*I might kick myself in the ass for saying this, but I might consider a mobile facility that, it’s not stationed in one place, but maybe they don’t have to be right on the main street… I’m opposed to this, but I would consider a mobile.* (Toronto resident)
5. Worry that a SIF(s) will renew problems that existed prior to neighbourhood gentrification	*[W]e had swarms of drug dealers and drug users, and we don’t want to go back there again* (Toronto resident)
6. Concern that resources for drug use prevention and treatment will be diverted to pay for SIFs	*And as a matter of fact, if it in any way compromised the integrity of things like methadone clinics, and other types of treatment options that are out there, I wouldn’t support it.* (Toronto health care worker)
	*…if they take away resources from other things. So it does depend.* (Ottawa city employee)
7. Concern that SIF implementation must include evaluation and community consultation, and explicit commitment to discontinue a SIF(s) in the event of adverse outcomes	*I would want some kind of assurance, some accountability on the part of the people who are running these, saying that give them a time, and if it’s not working, then they have to agree that we can sort of shut it down. Just to actually say a permanent site, I’m totally against it. If it was something on a temporary basis, on a contract basis, I would support it.* (Toronto resident)

Ambivalence was also linked to perceptions that SIF goals are too narrow and a comprehensive strategy for drug-related problems, which may include SIFs, is necessary. While some participants could be swayed towards accepting SIFs as part of a comprehensive strategy, there was no consensus about what “comprehensive” included. Suggestions ranged widely, from simply adding new SIF objectives (e.g., include abstinence) to multi-faceted approaches (e.g., increased services for drug use prevention, treatment, primary care, mental health, and housing). While reasons for ambivalence were generally the same in both cities, more Ottawa than Toronto participants spoke about concerns that a SIF would “siphon” away resources for drug treatment. Further, ambivalent stakeholders framed SIFs as a solution for cities said to have “devastating” drug problems (e.g., the Downtown Eastside in Vancouver, Canada) and that did not match the problems they knew about or experienced in their communities.

While ambivalent participants said that there might be a “right place” for SIFs, they did not suggest precise locations but rather excluded locations near their homes or businesses, often out of concern regarding damaging outcomes: e.g., “It's a good idea if it's not in my neighbourhood.” In neighbourhoods where gentrification was attributed to a reduction in local drug-related problems, some participants worried that a SIF(s) would renew these problems. A few ambivalent participants suggested that multiple or mobile SIFs would better address the dispersed nature of drug use in their city and alleviate concerns about congregation of drug users and drug dealing around a SIF(s).

Lastly, before offering their support to SIFs, ambivalent participants stated that they wanted assurances that any facility planning would include rigorous evaluation and opportunities for community consultation regarding SIF continuation or closure.

### Discussion

Our data show that community stakeholders who express ambivalence towards SIFs desire evidence about potential SIF impacts relevant to local contexts and that addresses perceived potential harms. These stakeholders would also prefer to see SIFs as part of a comprehensive drug strategy that ensures dedicated resource allocation for drug use prevention and drug treatment programs. During the focus groups, the moderators were often asked by participants to provide a rationale for SIFs given that needle and syringe programs (NSPs) are available in numerous locations in both Toronto and Ottawa (unpublished observations; Strike, Watson, and Kolla). These questions demonstrate lack of knowledge about the additional benefits of SIFs, over and above NSPs. Also, concerns that a SIF(s) would “siphon” away funds from drug treatment programs suggest lack of understanding of the ways in which SIF services can complement drug treatment programs [15].

There are several limitations to this study, including the purposive recruitment of participants. However, the aim of this sub-study was to complement existing reports of public opinion [7] with deeper exploration of stakeholders’ perspectives on SIF implementation. The sample size for this sub-study is large compared to other qualitative studies [16] and the repetition of reasons for ambivalence across focus groups and key informant interviews suggests that saturation was reached. These features add to the trustworthiness of the data. Nevertheless, our consultations took place in two cities without operational SIFs [9] and as such these results may not be generalizable to locations where SIFs have already been established. In future surveys of public opinion, including questions about the reasons for ambivalent opinions could provide representative estimates of such opinions and help estimate the relative importance of the reasons for ambivalence we outline above.

For those in favour of SIF implementation, an outstanding question for research and policy remains on the table – how might we better address ambivalence and encourage community stakeholders to support SIFs? A likely and important barrier to changing ambivalent opinions is continued stigma and discrimination towards people who use drugs and harm reduction services [17, 18]. Overcoming this stigmatization will take concentrated effort and broad community education. Making additional gains in SIF acceptance, in particular, appears to need ongoing processes of “policy mobilization” championed by local advocates and experts [19]. Our analysis offers a snapshot of reasons that may underlie SIF ambivalence that may ultimately help or hinder such processes in Canadian and international jurisdictions. The evidence we present is especially timely in Canada where very recently a bill, Bill C-2, that seeks to restrict the opening of new SIFs has received royal assent [20]. The bill outlines a lengthy list of criteria – with emphasis on broad community support – that must be met before the requisite legal exemption for a SIF can be granted [21, 22].

In short, we report evidence from a large, mixed-methods study designed by a group of multidisciplinary experts who received competitive, external funding for this research and who also benefited from access to a public opinion study [7]. These resources and the scope of our study are not easy to replicate. Other communities without access to the same level of scientific expertise and financial resources may face difficulties accumulating the breadth of data required under Bill C-2 or similar legislation that may exist elsewhere which, in turn, may hamper their ability to move forward with evidence-based harm reduction programs.

## Abbreviation

SIFs: Supervised injection facilities
